# High‐fat diet increases electron transfer flavoprotein synthesis and lipid respiration in skeletal muscle during exercise training in female mice

**DOI:** 10.14814/phy2.15840

**Published:** 2023-10-19

**Authors:** Philip M. Batterson, Erin M. McGowan, Agnieszka K. Borowik, Michael T. Kinter, Benjamin F. Miller, Sean A. Newsom, Matthew M. Robinson

**Affiliations:** ^1^ School of Biological and Population Health Sciences Oregon State University Corvallis Oregon USA; ^2^ Aging and Metabolism Research Program Oklahoma Medical Research Foundation Oklahoma City Oklahoma USA; ^3^ Oklahoma City VA Oklahoma City Oklahoma USA

**Keywords:** exercise, females, high‐fat feeding, mitochondrial respiration, skeletal muscle

## Abstract

High‐fat diet (HFD) and exercise remodel skeletal muscle mitochondria. The electron transfer flavoproteins (ETF) transfer reducing equivalents from β‐oxidation into the electron transfer system. Exercise may stimulate the synthesis of ETF proteins to increase lipid respiration. We determined mitochondrial remodeling for lipid respiration through ETF in the context of higher mitochondrial abundance/capacity seen in female mice. We hypothesized HFD would be a greater stimulus than exercise to remodel ETF and lipid pathways through increased protein synthesis alongside increased lipid respiration. Female C57BL/6J mice (*n* = 15 per group) consumed HFD or low‐fat diet (LFD) for 4 weeks then remained sedentary (SED) or completed 8 weeks of treadmill training (EX). We determined mitochondrial lipid respiration, RNA abundance, individual protein synthesis, and abundance for ETFα, ETFβ, and ETF dehydrogenase (ETFDH). HFD increased absolute and relative lipid respiration (*p* = 0.018 and *p* = 0.034) and RNA abundance for ETFα (*p* = 0.026), ETFβ (*p* = 0.003), and ETFDH (*p* = 0.0003). HFD increased synthesis for ETFα and ETFDH (*p* = 0.0007 and *p* = 0.002). EX increased synthesis of ETFβ and ETFDH (*p* = 0.008 and *p* = 0.006). Higher synthesis rates of ETF were not always reflected in greater protein abundance. Greater synthesis of ETF during HFD indicates mitochondrial remodeling which may contribute higher mitochondrial lipid respiration through enhanced ETF function.

## 1 INTRODUCTION

High‐fat diet (HFD) and exercise are robust stimuli to remodel the skeletal muscle mitochondria. Diets high in fat content have been shown to increase mitochondrial content (i.e., mitochondrial quantity) and respiratory capacity (Hancock et al., 2008; Leckey et al., 2018; Turner et al., 2007) with preferential regulation of fat oxidation. Indeed, HFD remodels the mitochondrial proteome toward higher abundance of proteins for fat oxidation (Dasari et al., 2018), alongside increased mitochondrial protein synthesis rates and increased maximal capacity for fat oxidation compared to low‐fat diet (LFD) (Ehrlicher et al., 2020; Newsom et al., 2017; Turner et al., 2007). These adaptations occur with no change in skeletal muscle respiratory capacity for non‐lipid substrates (Ehrlicher et al., 2020; Newsom et al., 2017). Exercise has been shown to increase mitochondrial content and respiratory capacity across both lipid and non‐lipid substrates (Holloszy, 1967; Jacobs et al., 2013; Robinson et al., 2017) through induction of gene pathways and protein synthesis associated with mitochondrial biogenesis and mitochondrial complex proteins (Batterson et al., 2023; Pilegaard et al., 2003; Robinson et al., 2017). Studies from our lab in male mice indicate that HFD and exercise can each increase skeletal muscle mitochondrial fat oxidation and increase maximal oxidative phosphorylation capacity (Ehrlicher et al., 2020; Newsom et al., 2017). In our previous work, HFD increased mitochondrial fat oxidation when normalized to mitochondrial protein content (Ehrlicher et al., 2020). The evidence suggests that HFD may be a more potent stimulus for mitochondrial remodeling of fat oxidation compared to exercise.

Oxidation of lipid requires electron transport from electron transfer flavoprotein (ETF) which is a distinct electron entry point into the electron transfer system independent of NADH. ETF is a heterodimeric inner mitochondrial membrane protein with alpha (ETFα) and beta (ETFβ) subunits that catalyzes β‐oxidation through the transfer of reducing equivalents to ubiquinone (Ramsay et al., 1987; Roberts et al., 1996). Changes to ETF enzyme function and protein content could be important to drive improvements in mitochondrial fat oxidation. Increases in lipid respiration in response to HFD in male mice are accompanied by increases in ETFα and ETFβ subunits (Ehrlicher et al., 2020). However, the mechanisms responsible for increased ETF protein in response to HFD and exercise remains to be fully elucidated.

Protein synthesis is a mechanism driving remodeling of mitochondrial proteins to HFD or exercise. Declines in skeletal muscle protein synthesis are observed in HFD‐induced metabolic diseases (Gonzalez et al., 2011; Møller et al., 2017; Nilsson et al., 2013) but can be restored with exercise training (Ehrlicher et al., 2020). Previous studies have shown that different mitochondrial compartments have different protein fractional synthetic rates (Ehrlicher et al., 2020; Wolff et al., 2021), that can be modified by diet or exercise (Ehrlicher et al., 2020; Nilsson et al., 2013). Turnover rates are protein specific (Jaleel et al., 2008) and HFD induces changes to the skeletal muscle mitochondrial proteome through greater changes to β‐oxidation than electron transfer proteins (Dasari et al., 2018). Therefore, differential regulation of protein content through changes to protein synthesis rate of ETF proteins could be important for the maintenance of mitochondrial protein abundance and respiration per unit in response to HFD and exercise.

The adaptive response to HFD (Casimiro et al., 2021) and exercise is sex specific (Scalzo et al., 2014). Female mice show enhanced ability to oxidize dietary lipid during transition to HFD (Oraha et al., 2022) suggesting greater capacity for lipid respiration compared to males (Miotto et al., 2018; Von Schulze et al., 2018). Compared to male mice, female mice maintain higher locomotor activity during early stages of HFD which could result in exercise training being a lower absolute stress (Casimiro et al., 2021), reducing the ability of exercise to further increase lipid oxidation in the context of HFD in female mice. The aim of the current study was to determine intrinsic mitochondrial remodeling for fat oxidation through ETF in female mice. HFD appears to be a greater stimulus for remodeling fat oxidation compared to exercise training and female mice may be less responsive to exercise. Therefore, we hypothesized that HFD would increase lipid respiration and be a greater stimulus to induce remodeling of ETF compared to exercise in female mice.

## 2 METHODS

### 2.1 Animals and diets

**FIGURE 1:**
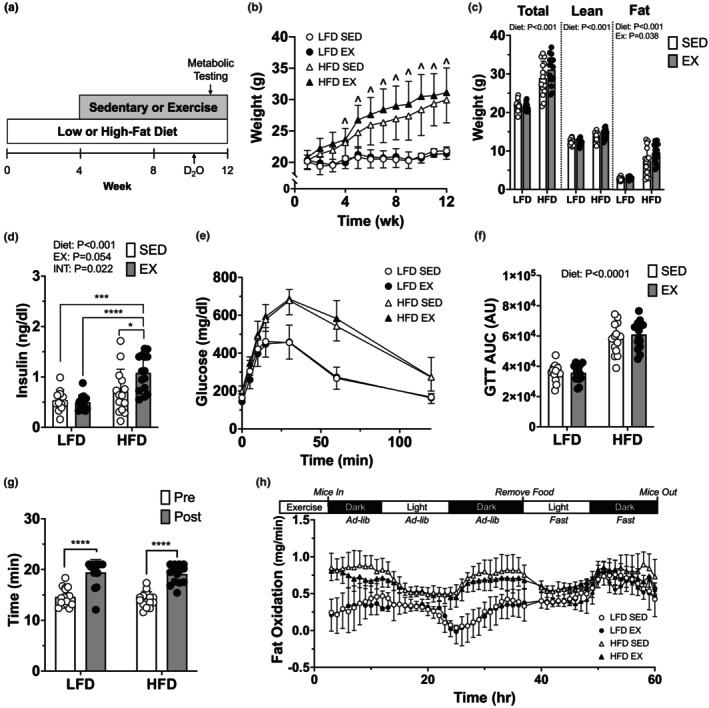
High‐fat diet induced weight gain, glucose intolerance, and reliance on whole‐body lipid oxidation but did not hinder their ability to adapt to exercise training. (a) study timeline. (b) body weight. (c) total mass with lean and fat mass (grams) after 12‐week intervention. (d) fasting plasma insulin concentrations taken on Week 11 before glucose tolerance test (GTT). (e) blood glucose response to intraperitoneal glucose injection, and (f), area under the curve. Blood glucose was measured at 0, 5, 10, 15, 30, 60, and 120 min. (g) time to exhaustion during graded exercise test (GXT) before and after training. (h) indirect calorimetry was used to calculate absolute fat oxidation rates with 1‐h averages. Data displayed as mean ± SD from *n* = 15 C57BL/6J female mice for the low‐fat diet (LFD) sedentary (SED), LFD exercise (EX), and high‐fat diet (HFD) SED groups, *n* = 14 for HFD EX. Effects of diet and exercise were evaluated using two‐way ANOVA. Graded exercise test time was analyzed with nonparametric Wilcoxon test. *p*‐values are reported when reaching statistical significance. When interactions were detected, Tukey post hoc paired comparisons were completed and reported such that * <0.05, ** <0.01, *** <0.001, **** <0.0001. ^ denote main effect of diet.

Wild‐type C57BL/6J female mice were purchased from Jackson Laboratories (Bar Harbor, ME, USA) and matched for age (8–14 weeks old). The mice were group housed five per cage in 12:12‐h light–dark cycle at 22°C with free access to food and water and allowed to acclimate for at least 1 week before beginning the 12‐week study. Mice were given a low‐fat diet (LFD; D12450J from Research Diets, New Brunswick, NJ, USA) or high‐fat diet (HFD, D12492 from Research Diets, New Brunswick, NJ, USA) for 4 weeks prior to the start of exercise (Figure 1a). After 4 weeks, mice began graded aerobic exercise training (EX) (described below) or remained sedentary (SED) while continuing their allotted diet. The percent kilocalories from total fat/carbohydrate/protein were 10/70/20 for the LFD (3.85 kcal/gm) and 60/20/20 for the HFD (5.24 kcal/ gm). The diets were matched for sucrose content.

In Week 12, mice were euthanized 36 h after the last bout of exercise and after a 4‐h fast to avoid alteration of autophagy markers by acute exercise, feeding, or prolonged fasting signals. Mice were anesthetized with isoflurane inhalation and cardiac punctures were performed to collect plasma samples. Tissues were extracted and dissected then snap frozen in liquid nitrogen. Mice were 24–28 weeks of age at tissue collection. The protocol was approved by the Institutional Animal Care and Use Committee at Oregon State University (#2019‐0003). Final group numbers were *n* = 15 for LFD SED, LFD EX, and HFD SED and *n* = 14 for HFD EX.

### 2.2 Exercise training

Exercise training was performed on a motorized treadmill to standardize the amount of exercise for each group to 50 min per day, 5 days per week as before in male mice (Ehrlicher et al., 2020). Mice in the exercise groups acclimated to the motorized treadmill (Panlab, Harvard Apparatus, Holliston, MA, USA) over 1 week with low speed and duration that was gradually progressed. Then, each exercise bout started with a warm‐up at 6 m/min for 5 min followed by exercise bouts that progressed from 10 m/min at 0% incline to 17 m/min for 50 min at 10% incline by Week 7 of training, the final 2 weeks were maintained at this exercise intensity. Mice were encouraged to continue running with an air puff or mild shock plate at the back of the treadmill.

A graded exercise test (GXT) was performed after the acclimation and at the end of the training period to assess exercise capacity of the mice. The belt speed started at 6 m/min and 0% incline for 5 min then increased by 3 m/min and 5% incline every 2 min until 18 m/min and 15% incline was reached. The speed increased by 1–2 m/min every minute until exhaustion. The mice were removed from the treadmill when they refused to run, despite sitting on the shock grid for up to 5 s, and the total time was recorded. The graded exercise test was stopped if mice were able to complete Stage 15 of the GXT (30 m/min at 15% incline).

### 2.3 Glucose and insulin tolerance tests

Glucose tolerance tests (GTT) and insulin tolerance tests (ITT) were performed in unrestrained mice at baseline and repeated at 4 weeks and 12 weeks. Mice were fasted and placed in individual cages with free access to water for 6 h prior to the GTT and 4 h prior to the ITT as described (Ehrlicher et al., 2020; McGowan et al., 2022; Newsom et al., 2017). Body weight was recorded after the fasting period to calculate injection volumes. Blood glucose concentrations were measured by a handheld glucometer (Alphatrak2, Zoetis, Parsippany, NJ, USA) from a tail vein slice. Blood samples were also collected into microhematocrit capillary tubes from the tail vein and centrifuged to separate plasma for insulin concentrations (Insulin ELISA, Alpco, Salem, NH, USA) immediately before and 15 min into the GTT. For the GTT, glucose was provided with an intraperitoneal injection of 2 g/kg body wt of a 20% dextrose solution. Blood glucose concentrations were measured at 0, 5, 15, 30, 60, and 120 min to calculate an area under the curve (AUC) for the full 2 h. For the ITT, insulin (Humulin R; Eli Lilly, Indianapolis, IN, USA) was provided with an intraperitoneal injection at 0.5 IU/kg body wt. Blood glucose was measured at 0, 15, 30, 60, and 120 min and fall from baseline (FFB) was calculated over the first 30 min.

### 2.4 Deuterium oxide labeling

In Week 10, mice received an intraperitoneal injection of deuterium oxide (D_2_O) saline solution (~99%; Cambridge Isotope Laboratories, Tewksbury, MA, USA) at 30 μL/g body weight to raise body water enrichment to ~5% (assuming 60% water weight) followed by continuous consumption of D_2_O enriched at 8% in the drinking water for the final 2 weeks of the study as previously described (Drake et al., 2015; Ehrlicher et al., 2020; Kobak et al., 2022; Newsom et al., 2017). Plasma samples collected at the time of euthanasia were analyzed for D_2_O enrichment to calculate precursor enrichment. For body water enrichment, 100 μL of plasma was placed in the inner well of an o‐ring cap of inverted screw‐capped tubes and placed in a heat block for overnight distillation at 80°C. Distilled samples were diluted 1:300 in ddH2O and analyzed on a liquid water isotope analyzer (Los Gatos Research, Los Gatos, CA, USA) against a standard curve prepared with samples containing different concentrations of D_2_O (Abbott et al., 2021; Borowik et al., 2023; Brown et al., 2021; Kobak et al., 2021).

### 2.5 Whole‐body metabolic assessment

Metabolic rate was assessed by indirect calorimetry using a continuous metabolic monitoring system (Promethion, Sable Systems Int., Las Vegas, NV, USA). Mice were singly housed in the metabolic cages immediately after an exercise bout and given ad libitum access to food and water for a 12‐h dark cycle to monitor whole‐body metabolism after exercise as previously described (Ehrlicher et al., 2020; McGowan et al., 2022). The mice remained in the cages with access to food for a full 24‐h light/dark cycle to measure metabolism in a rested condition. The food was removed, and mice remained in cages for 24 h to measure metabolism in the fasted state. Air was sampled periodically from each cage and passed through a gas analyzer to determine oxygen (O_2_) and carbon dioxide (CO_2_) content at a constant flow rate of 2000 mL/min. Energy expenditure and respiratory exchange ratio (RER) was calculated based on VO_2_ consumption and VCO_2_ production as either 1‐h or 12‐h averages for each cage. Body composition was measured by dual‐energy x‐ray absorptiometry (Lunar PIXImus2, GE Healthcare, Madison, WI, USA) to quantify fat and fat‐free (lean) mass.

### 2.6 Mitochondrial respiration

Mitochondria were isolated from fresh whole quadriceps samples then analyzed using high‐resolution respirometry (Oxygraph O2K, Oroboros Instruments, Innsbruck Austria). Approximately 100 mg of skeletal muscle was minced and incubated for 7 min on ice in 3 mL of buffer A (100 mM KCl, 50 mM Tris base, 5 mM MgCl2‐6H2O, 1.8 mM ATP, and 1 mM EDTA, pH 7.2) containing protease (Subtilisin A, Sigma P5380) then diluted in another 3 mL of buffer A and homogenized in glass‐on‐glass homogenizers with 0.3‐mm spacing between mortar and pestle for 10 min at 150 revolutions per minute. Samples were centrifuged for 5 min at 750*g* and 4°C then supernatant was removed and centrifuged for 5 min at 10,000*g* and 4°C to pellet the mitochondria. The supernatant was discarded, and the mitochondrial pellet was washed with buffer A and centrifuged 5 min at 9000*g* and 4°C. The final mitochondrial enriched pellet was resuspended in 1:4.2 (wt/vol) buffer B (180 mM sucrose, 35 mM KH_2_PO_4_, 10 mM Mg‐acetate, 5 mM EDTA, pH = 7.5).

High‐resolution respirometry was performed using Oxygraph‐2k units (Oroboros Instruments, Innsbruck, Austria) with MiR05 respiration buffer (0.5 mM EGTA, 3 mM MgCl_2_‐6H_2_O, 60 mM lactobionic acid, 20 mM taurine, 10 mM KH_2_PO_4_, 20 mM HEPES, 110 mM sucrose, and 1 g/L bovine serum albumin). Instrument settings were 37°C, stirring at 750 RPM and 2‐second data averaging (Datlab 7.4, Oroboros Instruments). We conducted two independent protocols to determine rates of oxygen consumption (JO_2_). Hydrogen peroxide (H_2_O_2_) emission was measured simultaneously using 10 μM Amplex red, 5 U/mL superoxide dismutase, and 1 U/mL horseradish peroxidase (calibrated with H_2_O_2_ injections) as previously reported and with a few variations (Batterson et al., 2023; Ehrlicher et al., 2020; McGowan et al., 2022; Newsom et al., 2017; Robinson et al., 2019). H_2_O_2_ production was used to calculate electron leak to H_2_O_2_ as the rate of H_2_O_2_ emission divided by two times the simultaneous rate of O_2_ consumption then multiplied by 100 for oxidative phosphorylation and leak respiration (Daussin et al., 2012). Each protocol was performed in duplicate chambers (A and B) on one machine and the rate of oxygen consumed (JO_2_ pmol O_2_/mL/sec) at each point was calculated as the average values from the two chambers. Protein concentration of the mitochondrial preparation was measured by Pierce BCA assay (23225 from Thermo Fisher Scientific, Waltham, MA) to calculate JO_2_ relative to protein content (pmol O_2_/μg protein/sec).

We used two separate titration protocols. The first titration sequence was addition of 50 μL of mitochondrial suspension then additions of 2 mM malate, 2.5 mM ADP, five titrations of 2 mM octanoylcarnitine to determine sensitivity (*K*_*m*_) and capacity (*V*_max_) of F‐linked substrates through ETF during oxidative phosphorylation (OXPHOS) to achieve saturating concentration of 0.5 mM, which aligns with previous protocols of fatty acid titrations below 60 μM (Petrick & Holloway, 2019), 100 mM ocantoylcarnitine, 20 μM cytochrome C (membrane integrity test), 2 μg/mL oligomycin (NS‐linked Leak), sequential additions of 0.5 μM FCCP to plateau (electron transfer capacity (E), F‐linked), 10 mM glutamate (FN‐linked E), 5 mM pyruvate (FN‐linked E), 10 mM succinate (FNS‐linked E), 1 mM glycerophosphate (FNSGp‐linked E), 0.5 μM rotenone (S‐linked E), and 2.5 μM antimycin A (residual oxygen consumption). A full representative tracing is in Figure S3. *K*_m_ and *V*_max_ were determined using the equation *Y* = *V*_max_*X/(*K*_*m*_ + *X*), where Y is the JO_2_ and *X* is concentration of titrated octanoylcarnitine using Graphpad Prism (9.4.1) (San Diego, California).

The second titration sequence was 50 μL of mitochondrial suspension, addition of 2 mM malate, 100 mM octanoylcarnitine (F‐linked leak), 2.5 mM ADP (F‐linked OXPHOS), 10 mM glutamate (FN‐linked OXPHOS), 10 mM succinate (FNS‐linked OXPHOS), 5 mM pyruvate (FNS‐linked OXPHOS), 1 mM glycerophosphate (FNSGp‐linked OXPHOS), 2 μg/mL oligomycin (FNSG_p_‐linked Leak), sequential additions of 0.5 μM FCCP to plateau (FNSG_p_‐linked E), 0.5 μM rotenone (S‐linked E), and 2.5 μM antimycin A (residual oxygen consumption). A representative tracing is in Figure S4. Membrane integrity of mitochondrial preparation was verified by determining stimulation of respiration after addition of cytochrome *c* (*p* > 0.05 for t‐tests between prior steady‐state respiration states with increases of +11% LFD SED, +16% LFD EX, +18% HFD SED, +12% HFD EX). Non‐mitochondrial respiration, determined by addition of antimycin a (complex III inhibitor), was <5% of maximal respiration.

### 2.7 Skeletal muscle protein abundance—Western blot

Our previous studies in male mice included western blotting to measure ETF and related proteins (Ehrlicher et al., 2020), so we used the similar methods in the current analysis of females mice to allow qualitative comparisons with previous studies. We then completed more in‐depth analysis using targeted proteomics for individual protein abundance and synthesis rates (described below in *Individual protein replacement rate and targeted proteomics*). Western blots are semiquantitative versus targeted proteomics being quantitative, so our intention was not to compare the two methods but to allow comparisons to previous findings while adding greater detail regarding turnover of individual subunits of ETF and related proteins.

Frozen muscle samples were homogenized as whole tissue (~40 mg of quadriceps) or mitochondrial isolations (~70 mg of gastrocnemius) for immunoblotting as described (Newsom et al., 2017). We previously reported, using proteomics, that the isolation yields a mitochondrial‐enriched fraction, but still has contamination of other fractions (Dasari et al., 2018). Immunoblotting of the myofibrillar, sarcoplasmic, and mitochondrial fractions previously demonstrated a high abundance of VDAC in the mitochondrial fraction with some contamination of tubulin (Ehrlicher et al., 2020). Mitochondrial‐enriched fractions for subsarcolemmal (SSM) and intermyofibrillar (IMFM) subpopulations were isolated through homogenization and differential centrifugation as before (Ehrlicher et al., 2020). Our previous proteomics assessment revealed mitochondrial isolations yield a mitochondrial enriched fraction, but has contamination of other fractions (Dasari et al., 2018). Protein concentration was determined using Pierce BCA assay (23225 from Thermo Fisher Scientific, Waltham, MA). Samples were diluted in Laemmli buffer, heated to 95°C for 5 min then 25–60 μg of protein were resolved on 10%–15% Bis‐Tris gels and transferred to nitrocellulose membranes. For whole‐tissue homogenate, a control sample was loaded at the left and right lanes of each gel to serve as an internal control and the average intensity of the control lanes were used to normalize band intensities between gels. Mitochondria isolations were analyzed on a single gel each for SSM and IMFM. Ponceau staining of membranes was performed to verify equal loading and transfer of protein to the nitrocellulose membrane. Membranes were blocked in 5% bovine serum albumin (BSA) in Tris‐buffered saline +1%Tween (TBST). Primary antibodies were diluted in 5% BSA‐TBST or 5% nonfat dry milk TBST and membranes incubated overnight at 4°C. Secondary antibodies were diluted in 5% BSA‐TBST or 5% nonfat dry milk‐TBST and membranes incubated at room temperature for 1 h. Primary antibodies were diluted 1:1000 and purchased from Abcam (Cambridge, United Kingdom) for OXPHOS cocktail (110413), ETFα (110316), ETFβ (240593), Trimethylated ETFβ (76118), ETFDH (103910), CPT1 (134988), PINK1 (23707), Mitochondrial fission factor (MFF) (81127); Cell signaling (Danvers, MA) for LC3 (12741), p62 (39749), Parkin (4211), and BNIP3 (3769), OPA1 (80471), mitofusion 2 (MFN2) (9482), Abcam, DRP1 (14647); Other primary antibodies included METTL20 (87995, Novus Biologicals, Littleton, CO), HADH (PA5‐28203, Thermo Fisher Scientific, Waltham, MA), and BCL2 (7382, Santa Cruz Biotechnology, Dalla, TX). Secondary antibodies were purchased from Licor (Lincoln, NE, USA), diluted 1:10,000 in 5% BSA‐TBST or 5% nonfat dry milk‐TBST and membranes incubated at room temperature for 1 h. Images were generated using infrared detection (Odyssey, Licor, Lincoln, NE) and analyzed using ImageView software (Licor, Lincoln, NE). Images were collected using a moderate‐intensity laser power without indication of oversaturation, indicating densitometry was performed within the wide linear range of the instrument. We purchased antibodies from commercial vendors and verified that primary antibodies produced bands at anticipated molecular weights.

### 2.8 Skeletal muscle real‐time PCR

Total RNA was isolated from ~20 mg of quadriceps using commercially available RNA extraction kits as per manufacturer description (RNeasy Mini Kit, Qiagen, Hilden, Germany). Total RNA was eluted in 50 μL of PCR grade water and concentration was quantified using a spectrophotometer (Nanodrop, Thermo Scientific, Waltham, MA) and integrity via 2100 Bioanalyzer (Agilent, Santa Clara, CA). Complementary DNA was prepared using the Taqman reverse transcription kit (Life Technologies, Agilent, Santa Clara, CA) per the manufacturer's instructions. Real‐time PCR was performed on a 7900HT Fast Real‐Time PCR System (Thermo Fisher Scientific, Eugene, OR, USA) using the Taqman gene expression assays. The following mouse gene expression assays (Thermo Fisher Scientific, Eugene, OR, USA) were used for quantitative real‐time PCR: ETFα (Mm00521254_m1 FAM), ETFβ (Mm07298608_m1 FAM), ETFDH (Mm01220210_m1 FAM), METTL20 (Mm01260699_m1 FAM), and LYRM5 (Mm00505001_m1 FAM). Reactions were performed in a 384‐well assay with a relative standard curve. All samples were plated in triplicate, and each plate contained one experimental gene and a control, Mouse 18s RNA (Mm03928990_g1). ETFα, ETFβ, and ETFDH were multiplexed while METTL20 and LYRM5 were single. Normalized intensity was determined from a relative standard curve. Fold‐change for each gene was assessed after normalization of intensity value to 18s RNA.

### 2.9 Individual protein replacement rate and targeted proteomics

Quadriceps muscle (~30–50 mg) were powdered in liquid nitrogen, diluted in 1:20 (wt/vol) in homogenization buffer (100 mm KCl, 40 mm Tris–HCl, 10 mm Tris Base, 5 mm MgCl2, 1 mm EDTA, 1 mm ATP, pH = 7.6) plus protease/phosphatase inhibitors (P8340, Sigma‐Aldrich, St. Louis, MO, USA) using a bead homogenizer (Southern Labware, GA, USA). Protein fractions were derivatized for analysis of deuterium enrichment in alanine using gas chromatography–mass spectroscopy (7890A GC‐Agilent 5975C MS, Agilent, Santa Clara, CA). To determine the precursor pool enrichment, plasma samples were prepared for analysis of deuterium enrichment on a liquid water isotope analyzer (Los Gatos Research, Los Gatos, CA, USA) using 0%–12% deuterium standards. The precursor enrichment of alanine was then adjusted by mass isotopomer distribution analysis. The deuterium enrichments of both the protein (product) and the precursor were used to calculate fraction new: Fraction new = E_product_/E_precursor_, where the E_product_ is the enrichment (E) of protein‐bound alanine and E_precursor_ is the calculated maximum alanine enrichment from equilibration of the body water pool as previously described (Abbott et al., 2021).

Protein samples for the targeted quantitative proteomics were prepared as previously described (Kinter et al., 2012; Kobak et al., 2022). The 80 μg of total protein were taken for analysis. All protein samples were spiked with 8 pmol BSA in 1% SDS as an internal standard. Next, the total protein was desalted by precipitation in 1 mL of acetone overnight at −20°C. The protein pellet was solubilized in 50 μL Laemmli sample buffer and 20 μg protein was run in a 12% SDS‐Page gel (BioRad Criterion system). Further gels were fixed, and proteins were stained with Coomassie blue (GelCode blue, Pierce Chemical Company). We used a standard in‐gel digestion method to obtained peptides (Kinter & Sherman, 2005; Kobak et al., 2022). Briefly, the gel pieces were washed to remove the Coomassie blue and then reduced in 10 mg/mL DTT, alkylated in 35 mg/mL iodoacetamide, and digested overnight with 1 μg trypsin per sample in 200 μL of 10 mM ammonium bicarbonate. The mixture of peptides was extracted from the gel, evaporated to dryness in a SpeedVac, and finally reconstituted in 150 μL 1% acetic acid (v/v) for LC‐tandem MS analysis.

Protein concentration and isotopic distribution were evaluated by LC–high‐resolution MS. We used a QEx Plus hybrid quadrupole‐orbitrap mass spectrometry system (ThermoScientific), a splitless nanoflow HPLC system with autoinjector (ThermoScientific), and a 10 cm C18 column (Phenomenex Aeris 3.6 μm Peptide XB‐C18 100A) packed in a fused silica electrospray tip (New Objective). About 5 μL sample volumes were injected and loaded onto the column at 1.5 μL/min with 0.1% formic acid. The column was eluted at 150 nL/min with a linear gradient of CH3CN in water with 0.1% formic acid (2% CH3CN to 65% CH3CN in 60 min). The orbitrap mass spectrometer acquired full scan mass spectra with a m/z resolution of 280,000. Ion source settings included a spray voltageof 1.5 kV, ion transfer tube temperatureof 300°C, and positive ions mode.

We monitored kinetics of individual proteins involved in mitochondria‐related biological processes, such as 12 Krebs cycle proteins, as well as 10 selected proteins specific to ETF and β‐oxidation. The high‐resolution accurate mass (HRAM) data were processed using Skyline software from the MacCoss laboratory (MacLean et al., 2010) to find and integrate the correct peptide chromatographic peak areas. Each analysis included at least two unique peptides from the target protein. The response for each protein was calculated as the total integrated area for all peptides monitored for that protein.

We used d2ome software to calculate synthesis rates of individual proteins (Sadygov et al., 2018). Briefly, the individual protein synthesis rates were calculated based on the mean values and pooled standard deviations of all monitored peptides for a target protein using the time course of deuterium incorporation and a nonlinear regression fit model (Kobak et al., 2022). To account for the impact of variability within quantifying peptides, d2ome‐implemented Grubbs' outlier detection and removal were used.

### 2.10 Statistics

The study was a two‐by‐two design to detect differences in diet and exercise. Primary outcomes were compared via two‐way ANOVA. Significant main effects of diet and exercise are reported. We analyzed mitochondrial respiration using two‐way ANOVA with repeated measures of respiratory substrates. We used fat‐free mass as a covariate when analyzing energy expenditure from metabolic cages (Tschöp et al., 2011). Tukey multiple comparisons were completed when interactions were detected. Graded exercise testing time was not normally distributed and analyzed with Wilcoxon test. Statistical significance was set at *α* = 0.05. Data are presented as mean and standard deviation as recommended in the field (Curran‐Everett, 2008), with individual data points displayed when possible. Power calculations are based on data published from our lab where lipid respiration normalized to mitochondrial protein content (JO_2_ in pmol/(s∙μg)) from HFD fed male mice with mean 4.22 and standard deviation of 0.90 (Ehrlicher et al., 2020). Using *n* = 15 per group, *α* = 0.05, and power (1‐β) of 0.5 and 0.8, an unpaired *t*‐test could detect differences of 0.67 and 0.95 JO_2_ pmol/(s∙μg), respectively.

## 3 RESULTS

### 3.1 High‐fat diet alters whole‐body metabolism in female mice

The purpose of the current study was to challenge the mitochondria with lipid overload in female mice and determine if moderate‐intensity exercise modified the effect of HFD. HFD‐fed mice were heavier than LFD mice (main effect of diet: *p* < 0.05) (Figure 1b) following the intervention. There was a main effect for diet such that HFD‐fed mice had greater lean and fat mass than LFD mice (Figure 1c). Exercised mice had greater fat mass than sedentary mice (main effect exercise: *p* < 0.05). The interaction of *diet* × *exercise* (*p* = 0.08) did not reach statistical significance for fat or fat‐free mass yet provides some evidence for interaction for greater total mass in HFD‐EX mice. Mice consuming HFD had higher fasting insulin than mice on LFD (main effect diet *p* < 0.001), while fasting plasma insulin was highest in HFD‐EX (*p* = 0.022 for *diet* x *exercise* interaction, Figure 1d). HFD increased area under the curve during the GTT compared to LFD (main effect of diet: *p* < 0.0001, Figure 1e and f). Exercise did not modify the response to diet on insulin tolerance during the ITT (no effect of exercise: *p* > 0.05, Figure S1b). Both LFD and HFD mice increased time to exhaustion during the post‐training GXT (*p* < 0.0001, Figure 1g). HFD had higher fatty acid oxidation following fed conditions (light and dark) indicating a higher reliance on fatty acid oxidation (main effect of diet: *p* < 0.0001, Figure 1h). Energy expenditure, with fat‐free mass as covariate, was highest in HFD‐SED mice (*p* = 0.003 for *diet* x *exercise*), which was most evident in dark‐feeding cycle and in fasted state (~12 h from food removed, *p* = 0.05 for *diet* × *exercise*). In the fasted state, the covariate of fat‐free mass had a positive main effect (*p* = 0.05) on energy expenditure which aligns with the positive relationship of mass to energy expenditure (Tschöp et al., 2011). Together, HFD‐induced weight gain, decreased glucose tolerance, and induced higher rates of fatty acid oxidation in female mice. Exercise resulted in rates of whole‐body energy expenditure alongside gains in fat mass, without major interaction between diet and exercise on the overall phenotype.

### 3.2 High‐fat diet alters mitochondrial respiration through increases to fat oxidation

**FIGURE 2:**
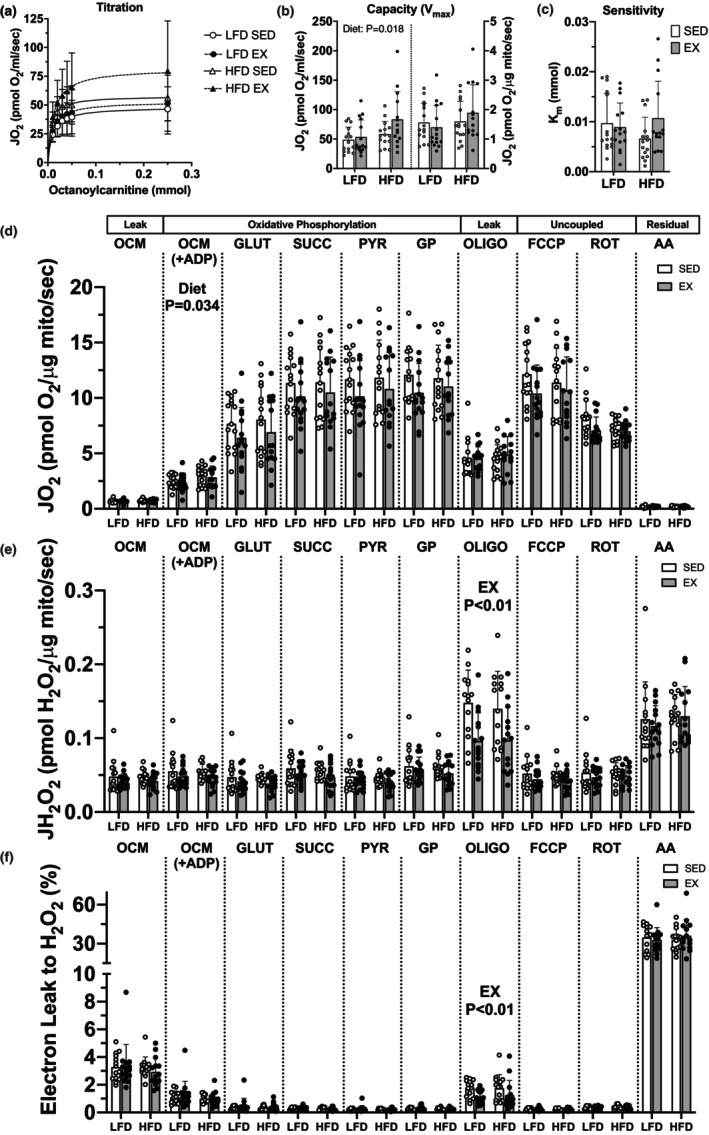
Twelve weeks of high‐fat diet but not exercise increased skeletal muscle mitochondrial capacity for lipid respiration. (a) titrations of octanoylcarnitine, (b) absolute and relative (to mitochondrial protein) capacity (*V*_max_), and (c) sensitivity (*K*_*m*_) for lipid respiration using titrations of octanoylcarnitine. (d) mitochondrial respiration during oxidative phosphorylation (in the presence of ADP) relative to mitochondrial protein abundance. (e) H_2_O_2_ emission during the respiration protocol. (f) electron leak to H_2_O_2_ production during respiration was calculated as the rate of H_2_O_2_ emission divided by two times the simultaneous rate of O_2_ consumption then multiplied by 100. Data displayed as mean ± SD. Effects of diet and exercise were evaluated within substrate using two‐way ANOVA (delineated with vertical lines with corresponding *p*‐value). We used two‐way ANOVA with repeated measures across substrates (OCM, OCM + ADP, GLUT, SUCC, PYR, GP) to detect differences over the titration. *p*‐values are reported when reaching statistical significance. Respiration rates are presented as absolute and normalized to mitochondrial protein abundance. Substrates used during titrations included: AA, antimycin A; ADP, adenosine diphosphate; FCCP, carbonyl cyanide‐p‐trifluoromethoxyphenylhydrazone; GLUT, glutamate; GP, glycerophosphate; OCM, octanoylcarnitine + malate; oligomycin, oligo; PYR, pyruvate; ROT, rotenone; SUCC, succinate.

We next explored the effect of HFD and exercise on skeletal muscle mitochondrial lipid respiration as mechanisms underlying the increase in whole‐body fatty acid substrate oxidation in response to HFD. We determined Michaelis–Menten kinetics for lipid respiration with titrations of octanoylcarnitine (Figure 2a). HFD increased absolute capacity (*V*_max_) for lipid respiration with no changes to sensitivity (*K*_*m*_) and exercise did not modify this effect (main effect of diet: *p* = 0.018, exercise: *p* = 0.07, Figure 2b,c). We performed a separate respiration experiment and used a two‐way ANOVA to determine respiration during oxidative phosphorylation (OCM + ADP through GP) with repeated measure of respiratory substrate. There was a main effect of exercise for greater respiration when expressed in absolute terms (*p* = 0.002, not shown). Normalizing respiration per mitochondrial protein reversed the main effect of exercise (e.g., lower with exercise, *p* = 0.08, Figure 2d) indicating a strong influence of mitochondrial abundance on respiration. We then used two‐way ANOVA (*diet* × *exercise*) to test individual responses of respiratory substrates, indicated in Figure 2d as vertical lines with corresponding *p*‐value for main effects. There was higher lipid respiration for HFD‐fed mice versus LFD when expressed per mitochondrial protein (diet effect *p* = 0.034). There were no effects of exercise on H_2_O_2_ emissions nor electron leak to H_2_O_2_ production during oxidative phosphorylation states (Figure 2e,f). Exercise trained mice had lower H_2_O_2_ emission and electron leak when measured during oligomycin‐induced leak (exercise effect *p* < 0.01 for H_2_O_2_ and electron leak). One mouse each from HFD + SED and LFD + SED had high H_2_O_2_ emission rates (>3× mean values) and are excluded from figures for clarity, yet including their data in models did not alter conclusions. The higher rates of electron leak to H_2_O_2_ with lipid substrates is consistent with previous reports of ETF being a site of reactive oxygen species production (Seifert et al., 2010). The diet effects on lipid respiration indicated increases to mitochondrial remodeling that were more specific to dietary fat composition, while the changes with exercise were more generalized across substrates.

### 3.3 High‐fat diet remodels fat oxidation through regulation of ETF pathway

**FIGURE 3:**
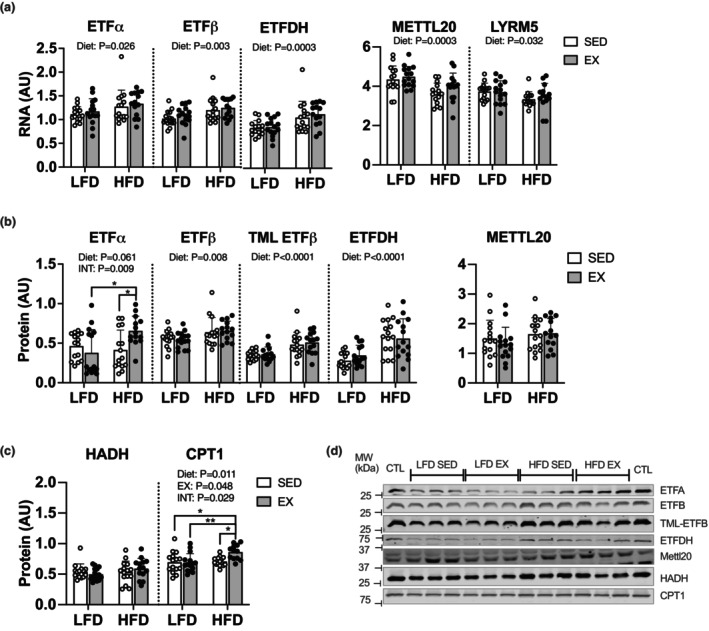
High‐fat diet remodeled the electron‐transferring flavoprotein pathway at the level of transcripts and protein. (a) RNA abundance via real‐time PCR. (b, c) protein abundance measured via western blot. (d), representative blots. Data displayed as mean ± SD. Effects of diet and exercise were evaluated using two‐way ANOVA. Images on representative blots come from separate membranes and channels. When interactions were detected, Tukey post hoc paired comparisons were completed and reported such that *<0.05, **<0.01.

**FIGURE 4:**
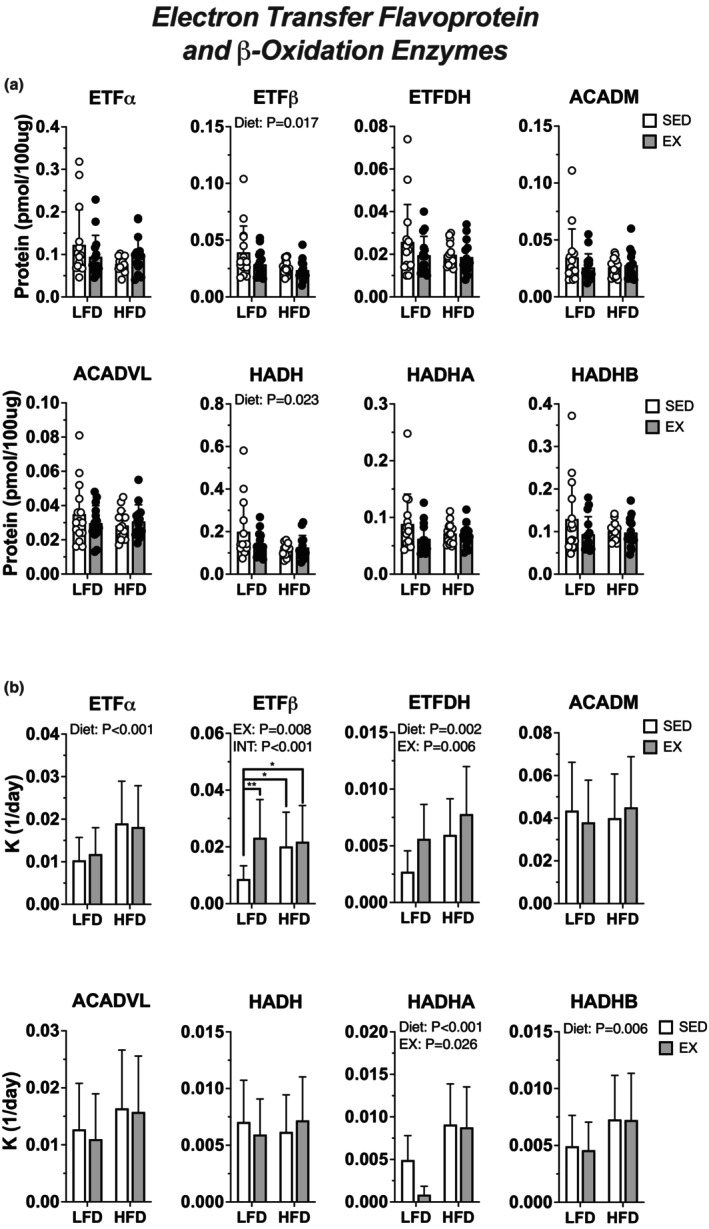
High‐fat diet increased synthesis of proteins within the electron‐transferring flavoprotein pathway. (a) protein abundance measured via targeted proteomics of quadriceps muscle. (b) protein synthesis rate constants, K, in % new protein per day. Data displayed as mean ± SD. Effects of diet and exercise were evaluated using two‐way ANOVA. When interactions were detected, Tukey post hoc paired comparisons were completed and reported such that * <0.05, ** <0.01.

We considered if the increases in lipid respiration could be explained by changes to transcriptional or translational regulation for markers of ETF and lipid transport and oxidation. HFD increased mRNA abundance of ETFα (*p* = 0.026), ETFβ (*p* = 0.004), and ETFDH (*p* = 0.003) and decreased mRNA abundance of METTL20 (*p* = 0.0003) and LYRM5 (*p* = 0.032) (Figure 3a). High‐fat diet increased protein abundance (measured via western blot) of ETFβ (*p* = 0.0084), trimethylated ETFβ (*p* < 0.0001), and ETFDH (*p* < 0.0001). There was an interaction between diet and exercise (*p* = 0.009) for exercising mice on HFD to have highest ETFα abundance (Figure 3b). There was no change to HADH protein abundance yet there was an interaction between diet and exercise for CPT1 abundance to be highest in exercising mice on HFD (interaction: *p* = 0.029, Figure 3c). Using quantitative proteomics identified no changes to most of the identified proteins for ETF, lipid transport and oxidation yet ETFβ and HADH were lower in HFD‐fed mice (Figure 4a). Thus, the effects of diet on abundance were modest and varied between individual protein subunits. We next measured protein synthesis by calculating the replacement rate constant (*K*) of proteins for ETF and lipid oxidation. Compared with LFD, there was a main effect of HFD for higher *K* for ETFα (*p* = 0.0007) and ETFDH (*p* = 0.0017). There was a main effect of exercise for higher *K* for ETFβ (*p* = 0.0079) and ETFDH (*p* = 0.0057). The effect of exercise was modified (an interaction) for ETFβ (*p* = 0.0332) (Figure 4b). Altogether, there was differential remodeling of mitochondrial fat oxidation during HFD and exercise. HFD stimulated remodeling of fat oxidation through induction of transcription, and induction of protein synthesis which may result in modest increases to protein abundance. Exercise had a small effect to modify protein synthesis for select ETF proteins.

### 3.4 High‐Fat diet decreased protein abundance for non‐lipid oxidation proteins with increased protein synthesis

**FIGURE 5:**
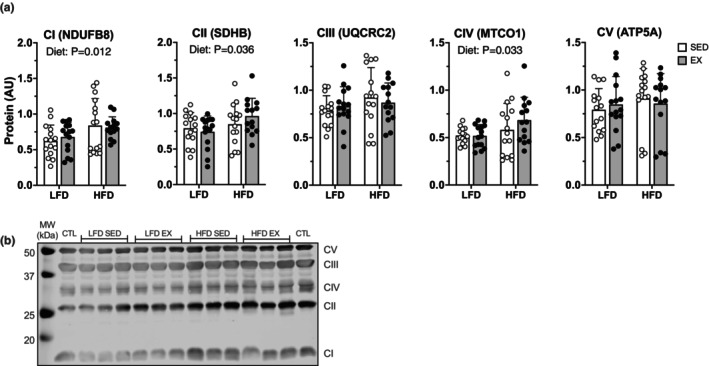
Twelve weeks of high‐fat diet increased protein abundance related to mitochondrial complexes without modification by exercise. (a) protein abundance in quadriceps muscle from female mice of mitochondrial complex subunit abundance measured via western blot and (b) representative blot. Data displayed as mean ± SD. Effects of diet and exercise were evaluated using two‐way ANOVA.

**FIGURE 6:**
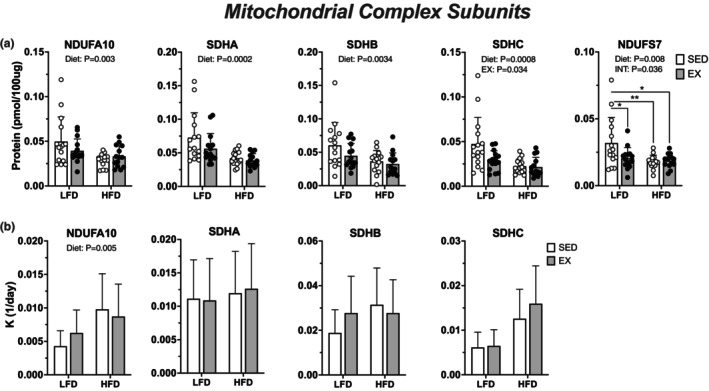
Twelve weeks of high‐fat diet decreased protein abundance related to mitochondrial complexes without modification by exercise. (a) protein abundance of mitochondrial complex subunits in quadriceps muscle from female mice measured via deuterium oxide labeled proteomics, with (b) protein synthesis rate constants. Data displayed as mean ± SD. Effects of diet and exercise were evaluated using two‐way ANOVA. When interactions were detected, Tukey post hoc paired comparisons were completed and reported such that *<0.05, **<0.01.

**FIGURE 7:**
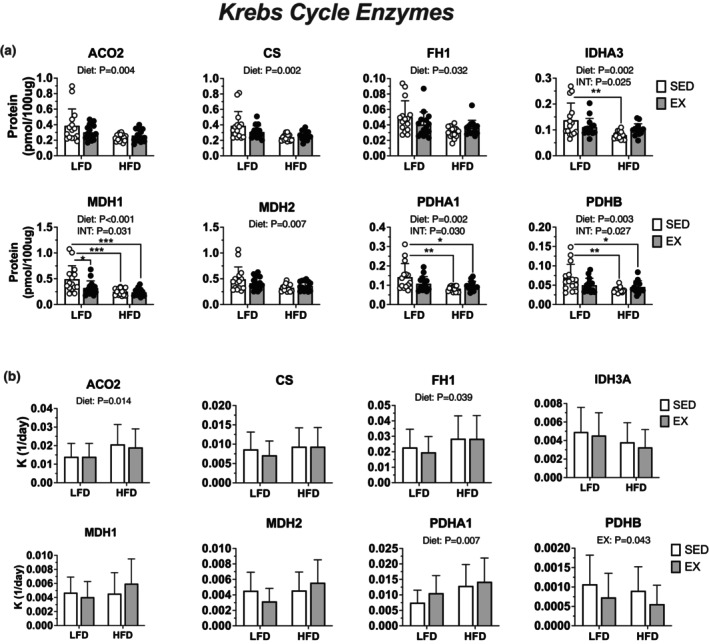
Twelve weeks of high‐fat diet increased protein synthesis related to Krebs cycle enzymes with subsequent decreases in protein abundance without modification by exercise. (a) protein abundance of Krebs cycle proteins in quadriceps muscle of female mice measured via deuterium oxide labeled proteomics, with (b), protein synthesis rate constants. Data displayed as mean ± SD. Effects of diet and exercise were evaluated using two‐way ANOVA. When interactions were detected, Tukey post hoc paired comparisons were completed and reported such that * <0.05, ** <0.01, *** <0.001.

We considered if lack of change in non‐lipid respiration could be explained by changes to translational regulation for markers of mitochondrial and non‐lipid oxidation proteins. Western blot analysis revealed a main effect of diet to increase protein abundance of CI [NDUFB8] (*p* = 0.012), CII [SDHB] (*p* = 0.036), and CIV [MTCO1] (*p* = 0.033) (Figure 5a). Targeted proteomics showed differential effects of HFD and EX on mitochondrial and Krebs cycle‐related proteins. There was a main effect of HFD to decrease protein abundance of 13 proteins measured while exercise had a main effect to decrease 1 protein (Figure 6a and 7a). There was an effect of HFD to increase K for 4 mitochondrial and Krebs cycle proteins (Figure 6b and 7b). The decrease in protein abundance for several Krebs cycle despite higher K indicates greater degradation of proteins could drive net loss of specific proteins during remodeling to HFD.

### 3.5 High‐Fat diet induces mitochondrial remodeling

**FIGURE 8:**
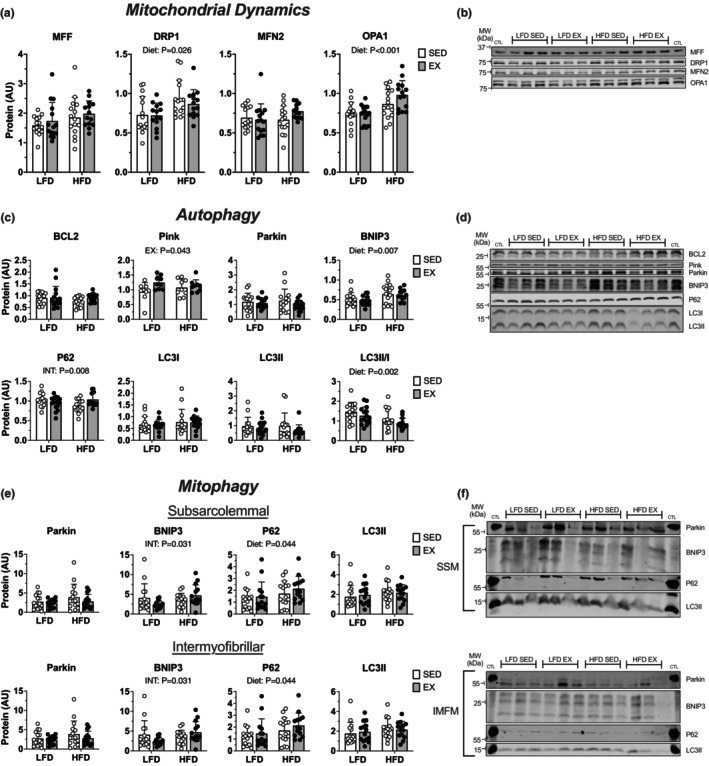
Twelve weeks of high‐fat diet induced mitochondrial remodeling through fission, fusion, and BNIP3‐dependent autophagy and mitophagy mechanisms. (a) markers of mitochondrial dynamics (fission and fusion) and (b) representative blots. (c) whole‐cell autophagy and (d) representative blots. (e) markers of mitophagy in mitochondrial subfractions (subsarcolemmal [SSM] and intermyofibrillar [IMFM]) and (f) representative blots. Whole‐cell lysates are from quadriceps and isolated mitochondria are from gastrocnemius. Data displayed as mean ± SD. Effects of diet and exercise were evaluated using two‐way ANOVA. Images on representative blots come from separate membranes and channels.

We considered if the overall changes to lipid and non‐lipid proteins could be explained by changes at the protein level for mitochondrial marker proteins of mitochondrial dynamics (Figure 8a), autophagy (Figure 8c), and mitophagy (Figure 8e). In whole‐cell lysate HFD animals had higher DRP1, OPA1, and BNIP3 abundance (main effect of diet: *p* = 0.026, *p* < 0.0001, and *p* = 0.007) and lower LC3II/I abundance (main effect of diet: *p* = 0.007). Exercised mice had higher Pink abundance (main effect of exercise: *p* = 0.043). There was an interaction of diet and exercise to modify P62 abundance (interaction *p* = 0.0075). Together these data indicate a modest induction of fusion, fission, and whole‐cell autophagy during HFD. Next, we considered if mitophagy was induced and measured proteins related to mitochondrial autophagy in mitochondrial subfractions. Within the subsarcolemmal mitochondria there was an effect of HFD to increase P62 abundance (main effect of diet: *p* = 0.044). There was an interaction of diet and exercise to modify BNIP3 abundance within both subsarcolemmal and intermyofibrillar mitochondria (*p* = 0.031 and *p* = 0.0043) (Figure 8e). Together, these data indicate a modest induction of autophagy targeted to mitochondria which may be mediated through a BNIP3‐dependent pathway.

## 4 DISCUSSION

Our purpose was to investigate ETF abundance and turnover in female mice as a mechanism contributing to changes in mitochondrial fat oxidation to HFD and exercise. HFD increases lipid respiration in males (Ehrlicher et al., 2020), which often have lower respiration than females (Cardinale et al., 2018; McGowan et al., 2022; Miotto et al., 2018; Montero et al., 2018). We studied female mice to determine if lipid respiration can be further stimulated amidst a background of higher intrinsic lipid respiration. We hypothesized that HFD would increase lipid respiration and be a greater stimulus to induce remodeling of ETF compared to exercise in female mice. We determined that HFD increased capacity for mitochondrial lipid respiration when expressed in absolute, and relative to mitochondrial protein abundance. Increased mitochondrial lipid respiration was accompanied by increases to turnover of ETF pathway components resulting in maintenance of protein abundance. Moderate‐intensity aerobic exercise had limited impact on skeletal muscle respiratory function or remodeling of ETF pathway components. Compared to lipid‐related proteins, HFD fed animals, had lower abundance of non‐lipid respiration proteins. Greater protein turnover of mitochondrial and Krebs cycle proteins could result in a net decrease in protein abundance. The primary findings indicate that HFD differentially remodels mitochondrial respiration for lipid respiration compared to non‐lipid respiration in female mice.

In female mice, HFD increased skeletal muscle absolute capacity for mitochondrial lipid respiration and relative capacity for oxidative phosphorylation of lipid substrates. These increases in respiration were accompanied by remodeling of ETF pathway proteins through increased transcription and protein synthesis resulting in a modest decrease to no change in protein content. The ETF is an inner mitochondrial membrane protein that accepts electrons from numerous acyl‐coA dehydrogenases (Watmough & Frerman, 2010) which are passed to ubiquinone, effectively joining β‐oxidation to direct electron entry to the electron transfer system (Ramsay et al., 1987; Roberts et al., 1996). Deficiencies and genetic mutations in ETF can result in decreased mitochondrial lipid respiration, and dissipation of membrane potential in cells (Chokchaiwong et al., 2019) alongside accumulation of acyl‐carnitines. Declines in acyl‐carnitines also occur during HFD‐induced obesity and are associated with decreased insulin sensitivity (Schiff et al., 2006). However, the regulation of ETF in response to HFD and exercise remains incomplete. Previous studies in male mice have shown increased lipid respiration with increases to protein content (measured via western blot) of ETFα and ETFβ following both HFD and exercise (Ehrlicher et al., 2020) similar to our western blot data presented in Figure 3.

The adaptive response to HFD (Casimiro et al., 2021) and exercise is different between males and females (Scalzo et al., 2014). Females have higher inherent β‐oxidation protein content and capacity for lipid respiration compared to males (Cardinale et al., 2018; McGowan et al., 2022; Miotto et al., 2018; Montero et al., 2018), which could limit the adaptive response to HFD and further increases to fat oxidation with exercise training. Indeed, in male mice, both HFD and aerobic exercise training increased lipid respiration and ETF protein abundance (Ehrlicher et al., 2020) whereas in our current study HFD, but not exercise, stimulated increased mitochondrial lipid respiration alongside greater protein synthesis indicating remodeling of mitochondria to diet. Compared to male mice, female mice have been shown to be resistant to weight gain during early HFD overfeeding (Casimiro et al., 2021; Oraha et al., 2022) and have enhanced reduction in respiratory quotient during HFD and western diet indicating enhanced ability to oxidize dietary lipid during early high‐fat challenges (McGowan et al., 2022; Oraha et al., 2022). A study comparing the effects of 20 weeks of HFD in male and female C57BL/6J mice showed that male mice were more susceptible to weight gain within 2 weeks of starting HFD, which was accompanied by reduced locomotor activity (Casimiro et al., 2021). Reduced activity could lead to exercise training being more of a stimulus for male compared to female mice, which may contribute to robust exercise responses in male but not female mice (Ehrlicher et al., 2020; McGowan et al., 2022). The females in our current training protocol improved performance during graded exercise tests, yet it is possible that lower absolute work in females or lack of robust training stimulus may contribute to why there were no major changes in glucose homeostasis nor body composition.

Increased absolute and relative (to mitochondrial protein content) lipid respiration in response to HFD alongside active transcriptional and translational regulation suggests HFD increased ETF enzyme function which could be contributing to increased lipid respiration. The mitochondrial protein pool is maintained by continuous protein synthesis and degradation. Increased protein synthesis rates could be one mechanism explaining higher ETF enzyme function following HFD in females. Mitochondrial protein synthesis has been shown to be increased in obese humans (Halvatsiotis et al., 2002) and rodents (Chanseaume et al., 2007; Newsom et al., 2017) although these findings are mixed (Beals et al., 2016; Guillet et al., 2009). Different mitochondrial compartments have different protein fractional synthetic rates (Ehrlicher et al., 2020) that can be modified with diet (Ehrlicher et al., 2020; Nilsson et al., 2013). Furthermore, previous studies have shown variable fractional synthesis rates for individual skeletal muscles (Stead et al., 2020) and individual mitochondrial proteins (Jaleel et al., 2008; Karunadharma et al., 2015; Wolff et al., 2021). Here we show that HFD, but not exercise training, increased protein synthesis rates of individual proteins for ETF and lipid respiration. However, HFD showed modest increase in protein synthesis rates of individual mitochondrial proteins that were not specific to lipid respiration. Increased protein synthesis rates for lipid‐related proteins could be contributing to remodeling without major changes in protein abundance.

The activation of degradation pathways and turnover of proteins is a possible mechanism to promote function in part through lowering damage to proteins. One suggested mechanism for increased protein damage associated with obesity is an increase in reactive oxygen species (ROS) specifically H_2_O_2_ emissions (Konopka et al., 2015). Increased mitochondrial ROS production has been observed in women with obesity and polycystic ovary syndrome which is reversed after exercise training (Konopka et al., 2015). We did not measure any overt derangement in ROS production in female mice, but electron leak to H_2_O_2_ production was ~3× higher for lipid substrates compared to other substrates. A consideration is that the absolute H_2_O_2_ production rates were similar across substrates, so changes in the relative portion of respiration due to electron leak to ROS was influenced by the varied O_2_ consumption rates between substrates. Higher absolute rates of ROS, not necessarily relative rates, can contribute to oxidative stress. A higher relative portion of respiration going to ROS may mean that at the same given oxygen consumption in vivo, HFD fed mice could be experiencing greater lipid flux into the mitochondria resulting in excess ROS production and greater oxidative damage (Muoio & Neufer, 2012). ROS can stimulate mitochondrial depolarization and activate mitophagy through Parkin‐dependent mechanisms (Wang et al., 2012). BNIP3‐dependent autophagy activation has been suggested as a means of quenching ROS production (Sowter et al., 2001) through HIF‐1 signaling (Sousa Fialho et al., 2021). We have shown that HFD and exercise regulate BNIP3 protein abundance in mitochondrial fractions and whole‐cell lysate (Ehrlicher et al., 2020; Ehrlicher et al., 2021) while others reported decreased BNIP3 abundance in response to HFD and western diet (Rosa‐Caldwell et al., 2017) which is rescued after exercise training (Cheng et al., 2022). Dysfunctional HIF‐1 signaling in cardiac tissue (an inability to stabilize HIF‐1α) has been implicated in the development of insulin resistance (Dodd et al., 2018), which suggests that HIF‐1 signaling and BNIP3 activation remain intact thereby removing oxidative damage caused during HFD induced obesity in female mice resulting in little to no mitochondrial defects. Our data support a modest increase in markers of autophagy activation in a BNIP3‐dependent manner after HFD in female mice which is not modified by exercise. The induction of protein synthesis and degradation help promote mitochondrial respiration.

In female mice, HFD increased absolute and relative mitochondrial lipid respiration without modification by exercise. Our previous studies of male mice have shown increased absolute and relative lipid respiration to both HFD and exercise (Ehrlicher et al., 2020). The change in relative lipid respiration indicates increased function per unit of enzymes across steps of lipid oxidation yet may range across β‐oxidation through the electron transfer system. ETF activity has been shown to be regulated by METTL20 via inhibition by trimethylation of the ETFβ subunit and is important during fasting and ketogenic conditions (Shimazu et al., 2018). Here we show that trimethylation of ETFβ increased after HFD and was not modified in response to training, suggesting that other mechanisms including enhanced ETF enzyme function may be responsible for increased lipid respiration following HFD in females.

The rates of mitochondrial lipid respiration were much lower than non‐lipid respiration. The lower rates for lipid respiration compared to non‐lipid respiration in the context of HFD‐induced lipid overload could be a contributing factor to the regulation and accumulation of bioactive lipid intermediates and subsequent inhibition of insulin signaling when substrate competition is present (Song et al., 2020). Furthermore, increased potential for ROS generation of lipid compared to non‐lipid intermediates could also contribute to diminished insulin signaling during HFD (Muoio & Neufer, 2012; Neufer, 2018). This inhibition of insulin signaling through increases to bioactive lipid intermediates and ROS production could result in metabolic inflexibility where cells are no longer able to switch between lipids and carbohydrates for generation of ATP (Song et al., 2020). Mitochondrial lipid respiration during OXPHOS increased in response to HFD but not after exercise. There was no change in respiration for any other substrates during OXPHOS and H_2_O_2_ emission did not change in response to either HFD or exercise training. For non‐lipid substrates, during uncoupled respiration, continued additions of substrates for independent electron entry into the electron transfer system (ETS) resulted in continually higher respiration while continued addition of substrates during OXPHOS resulted a plateau in respiration (Figure S2). These titration protocols with multiple substrates suggest that, in isolated mitochondria, electron flow through the electron transfer system exceeds respiration linked to ATP synthase. The higher respiration for complex I (e.g., NADH‐linked) respiration versus lipid substrates (which also generate NADH in β‐oxidation) indicates that complex I capacity does not limit lipid respiration. Previous studies have shown excess ETS capacity during lipid+non‐lipid supported complex I respiration in myocardial fibers (Lemieux et al., 2011) and complex I + II supported respiration in human permeabilized skeletal muscle fibers (Jacobs et al., 2013), although excess ETS is not always observed in isolated mitochondrial preparations (Newsom et al., 2017). We independently measured respiration during coupled and uncoupled substrate titrations and found an excess ETS capacity of about 37% after cumulative addition of substrates for lipid, CI, CII, and glycerophosphate, which is in line with previous studies from our lab (Ehrlicher et al., 2021). Electron entry of reducing equivalents from lipids into the mitochondria, either direct from β‐oxidation to ETF or via NADH to complex I, does not appear to be limiting for oxidative phosphorylation of lipids.

### 4.1 Study considerations

In humans, our lab (Batterson et al., 2023) and others (Larsen et al., 2015) have shown increased *V*_max_ for lipid substrates in lean and obese individuals in response to exercise training. These differences could be due in part to lipid overload from HFD being a specific nutrition stimulus for lipid oxidation compared to the moderate‐intensity exercise completed by the female mice in the current study. We did not include males, but a qualitative comparison reveals male mice from our previous studies had greater lipid oxidation capacity in response to the same exercise regimen (Ehrlicher et al., 2020). Female mice show enhanced ability to oxidize dietary lipid during transition to HFD (Oraha et al., 2022) suggesting greater capacity for lipid respiration compared to males (Miotto et al., 2018; Von Schulze et al., 2018). Estrogen signaling contributes to mitochondrial dynamics (Ribas et al., 2016). We did not control for estrous cycle and expect that effects were distributed across groups. Female mice also maintain higher locomotor activity during early stages of HFD which could result in exercise training being a lower absolute stress (Casimiro et al., 2021), reducing the ability of exercise to further increase lipid oxidation and subsequent decreases in systemic glucose homeostasis in the context of HFD in female mice. We measured protein abundance for lipid and non‐lipid oxidation proteins using western blot in both studies but now add targeted proteomics. We used western blot to allow comparison with previous work and interpret changes to protein abundance between female mice and male mice (Ehrlicher et al., 2020). The in‐depth quantitative analysis using targeted proteomics and synthesis rates from the same muscle group can determine individual protein dynamics. Based on the quantitative proteomics findings, we interpret that non‐lipid proteins appear to be more highly degraded in response to HFD while abundance of lipid‐related proteins is maintained through increased protein synthesis. Our steps to promote consistent isolation of mitochondria include that similarly trained technicians isolated mitochondria from the same general starting weight of tissue and diluted final pellets in buffer based on wet tissue weight (~95–105 mg of quadriceps). Furthermore, we measured mitochondrial protein content from each aliquot of isolated mitochondria that was added to the respiration chambers to calculate relative respiration rates. Autophagy inhibitors are needed to determine dynamics of flux. Our use of isolated mitochondria provides indication of autophagy and mitochondrial dynamics. However, we did not use treatment with an autophagy inhibitor in mice (i.e., chloroquine) because our primary focus was on synthesis rates, thus limiting interpretation of autophagy and mitochondrial degradation. We used quadriceps muscle for mitochondrial respiration and measures of whole‐cell lysate (i.e., mitochondrial complexes, dynamics, and autophagy) then used gastrocnemius subsarcolemmal and intermyofibrillar mitochondrial blots. We attempted to minimize influence of fiber type by using mixed fiber types (e.g., red and white heads of gastrocnemius and mixed quadriceps) yet cannot exclude that fiber type differences may influence interpretation between tissues types. The titration protocols for Figure S2 had different additions (i.e., succinate was added before pyruvate in the oxidative phosphorylation protocol). Our purpose in that experiment was to determine respiration during continued additions of electron donors during coupled and uncoupled states. Our main outcome was oxidative phosphorylation and, as glutamate can saturate NADH‐supported respiration in isolated mitochondria, we did not add pyruvate as another NADH source and proceeded directly to succinate. During uncoupled respiration, we added pyruvate prior to succinate to determine if respiration would increase amidst unrestricted electron flow. Four out of five states had overlapping substrates between the runs and during those states, the stepwise increases for uncoupled respiration was larger than during respiration coupled to ATP production. It is possible that identical titration would have stepwise increase in coupled respiration prior to succinate, yet the small change from succinate to glycerophosphate indicates that respiration coupled to oxidative phosphorylation may become saturated as compared to uncoupled electron flow. We used isolated mitochondria for respiration experiments because the approach samples a large portion of muscle (e.g., ~100 mg starting tissue) that is less susceptible to the influence of muscle heterogeneity from smaller sampling approaches. Previous studies demonstrate that isolation procedures alter mitochondrial morphology as compared to permeabilized fiber bundles (Picard et al., 2011). Our methods may therefore bias sampling toward mitochondria that can sustain processing and make it challenging to directly relate respiration to protein abundance in whole‐cell lysates or extrapolate to in vivo bioenergetics.

## 5 CONCLUSIONS

In conclusion, we show that increases to skeletal muscle lipid respiration after HFD in female mice is accompanied by lipid‐specific mitochondrial remodeling through transcriptional and translational regulation of ETF. Increases to protein turnover of ETF pathway proteins could drive higher enzyme function and contribute to increased lipid respiration following HFD. Together these findings demonstrate female mice adapt to HFD‐induced obesity through responsive induction of remodeling of lipid respiration pathways which could represent an important differential regulation point compared with male mice. Future studies should investigate the effects of more intense exercise on the remodeling of mitochondrial respiration and individual protein turnover between sexes.

## AUTHOR CONTRIBUTION

All authors contributed to conception/design of the work, acquisition, analysis or interpretation of data for the work and drafting the work or revising it critically for important intellectual content. All authors approved the final version of the manuscript; agree to be accountable for all aspects of the work in ensuring that questions related to the accuracy or integrity of any part of the work are appropriately investigated and resolved; and all persons designated as authors qualify for authorship, and all those who qualify for authorship are listed.

## FUNDING INFORMATION

The authors thank funding sources for support of the project including the Medical Research Foundation from Oregon Health Sciences University to MMR (2020‐2873), Nathan Shock Pilot Grant to MMR (NIH P30 AG050911), and Oregon State University for Provost Fellowship to PMB.

## CONFLICT OF INTEREST STATEMENT

The authors report no competing interests.

## ETHICS STATEMENT

The study was approved by the Institutional Animal Care and Use Commitee at Oregon State University (#2019‐0003).

## Supporting information


Figure S1–S4.

Data S1.

## Data Availability

The authors confirm that the data supporting the findings of this study are available within the article.
