# Supplementary material for: High‐fat diet increases electron transfer flavoprotein synthesis and lipid respiration in skeletal muscle during exercise training in female mice
Source: Physiol Rep. 2023 Oct 19;11(20):e15840. doi: 10.14814/phy2.15840 (PMC10587055; doi:10.14814/phy2.15840)
Supplement: Supplementary file 1 — Figure S1–S4. [file PHY2-11-e15840-s001.docx]

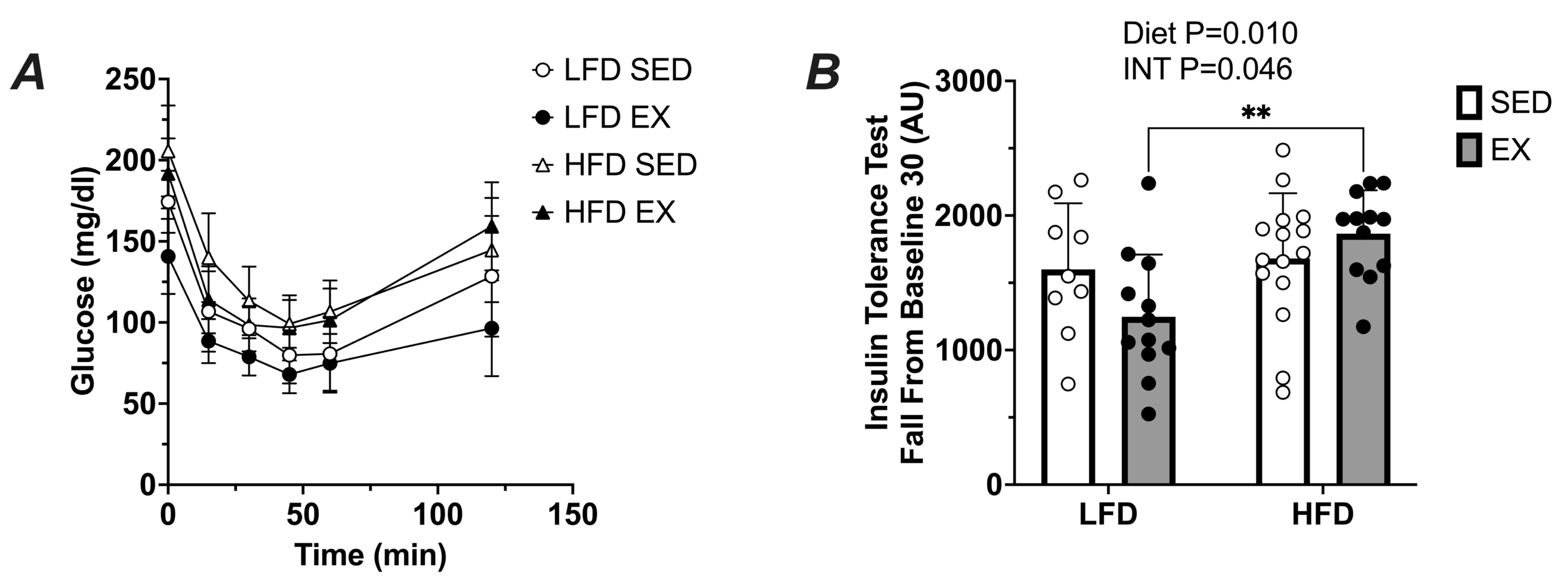


**Supplemental Figure 1: Response to insulin tolerance test (ITT) at week 12.** A, plasma glucose response over 120 minutes following insulin injection. B, ITT plasma glucose fall from baseline to 30 minutes. Data displayed as mean±SD. Data are from n=9 C57BL/6J female mice for the low-fat diet (LFD) sedentary (SED), n=12 LFD exercise (EX), and n=15 high-fat diet (HFD) SED groups, n=12 for HFD EX. Effects of diet and exercise were evaluated using 2-way ANOVA. When interactions were detected, Tukey post hoc paired comparisons were completed and reported such that ** <0.01.


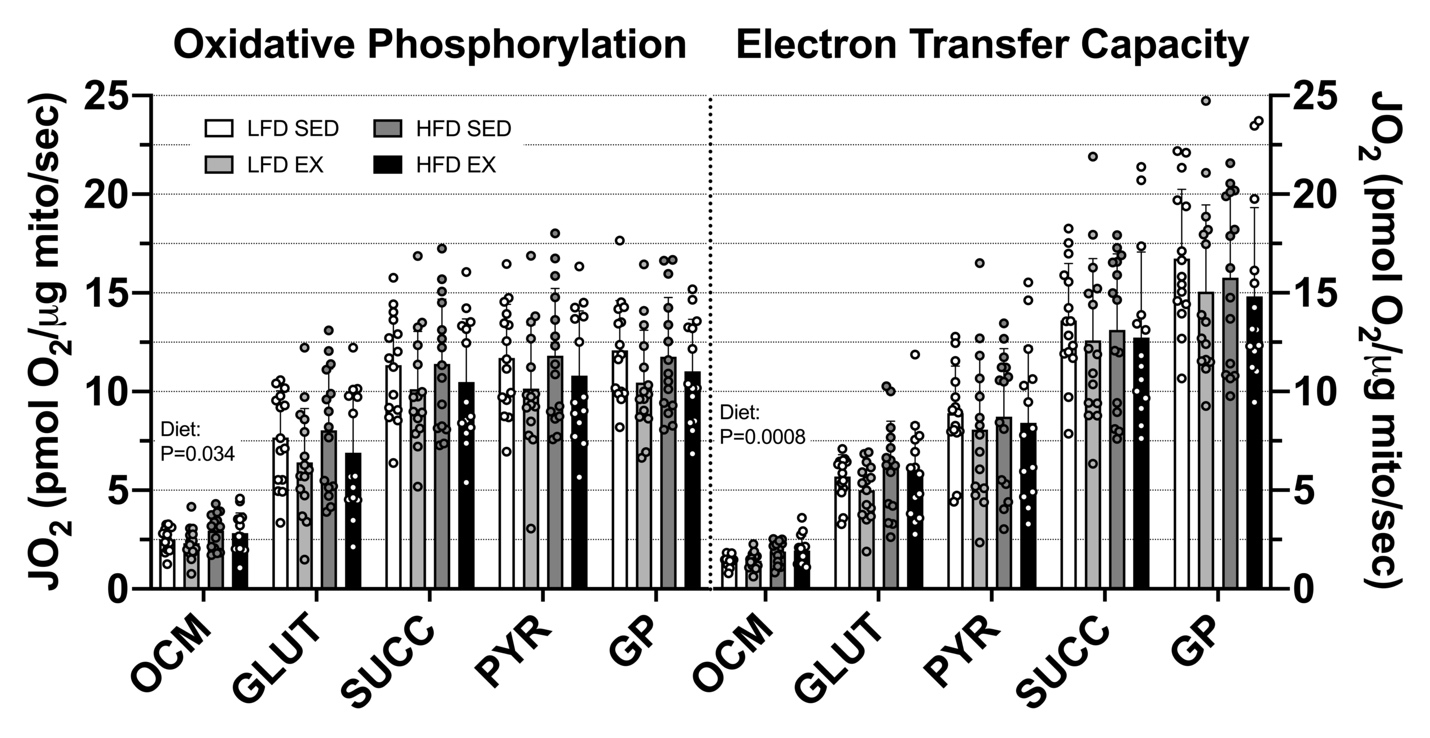


**Supplemental Figure 2: Electron transfer capacity continually increased with further additions of distinct electron donors while oxidative phosphorylation became saturated.** Separate titration protocols to measure respiration per mitochondrial protein for oxidative phosphorylation and electron transfer capacity (uncoupled through addition of FCCP). Additions included saturating octanoylcarnitine (OCM), glutamate (GLUT), succinate (SUCC), pyruvate (PYR) and glycerophosphate (GP) for OXPHOS and additions included saturating octanoylcarnitine (OCM), glutamate (GLUT), pyruvate (PYR), succinate (SUCC), and glycerophosphate (GP). Data displayed as mean±SD. Data are from n=15 C57BL/6J female mice for the low-fat diet (LFD) sedentary (SED), LFD exercise (EX), and high-fat diet (HFD) SED groups, n=14 for HFD EX. Effects of diet and exercise were evaluated using 2-way ANOVA. Respiration rates are normalized to mitochondrial protein abundance.

**Supplemental Figure 3:** Representative trace of octanoylcarnitine titration and electron transfer capacity protocol (uncoupled respiration). Additions included titration of malate (HMAL), adenosine diphosphate (HADP), sub-saturating octanoylcarnitine (OCM), high octanoylcarnitine (HOCT), cytochrome C (CYTC), oligomycin (OLIGO), FCCP, glutamate (GLUT), pyruvate (PYR), succinate (SUCC), glycerophosphate (GP), rotenone (ROT), and antimycin A (AMA).

**Supplemental Figure 4:** Representative trace of oxidative phosphorylation protocol (in the presence of ADP). Additions included titration of high octanoylcarnitine (HOCT), malate (HMAL), adenosine diphosphate (HADP), glutamate (GLUT), succinate (SUCC), pyruvate (PYR), glycerophosphate (GP), oligomycin (OLIGO), FCCP, rotenone (ROT), and antimycin A (AMA).
