# Supplementary material for: High‐fat diet increases electron transfer flavoprotein synthesis and lipid respiration in skeletal muscle during exercise training in female mice
Source: Physiol Rep. 2023 Oct 19;11(20):e15840. doi: 10.14814/phy2.15840 (PMC10587055; doi:10.14814/phy2.15840)

Representative Blot Images and Ponceau Stain

Full representative blot images and ponceau stains from western blot data in Figure 3B and C, 5A, and 8. Red boxed areas indicate regions displayed in manuscript.

A) ETF and Lipid Panel – Figure 3B and C

ETFA – Blot MRF9 ~35kDa 800 Channel

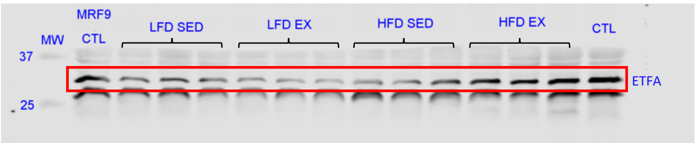

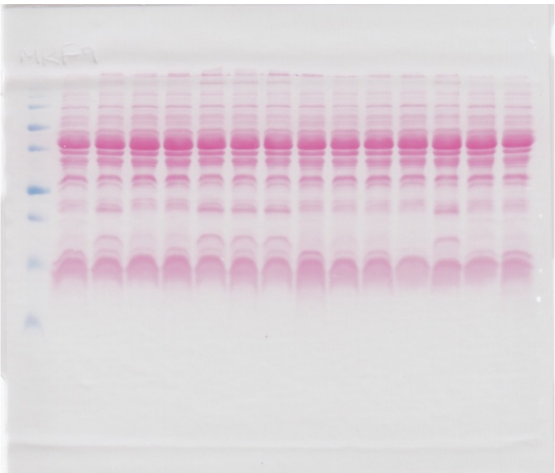

ETFB – Blot MRF9 ~30kDa 800 Channel

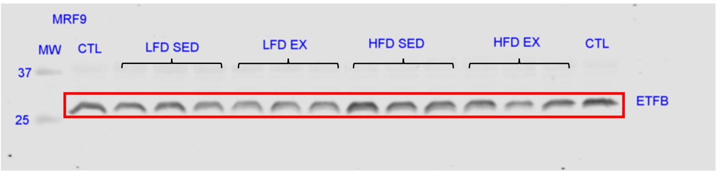

Trimethyl-ETFB – Blot MRF9 ~30kDa 700 Channel

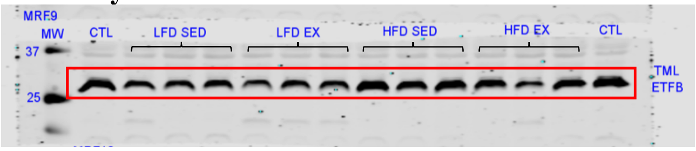

ETFDH – Blot MRF9 ~68kDa 800 Channel

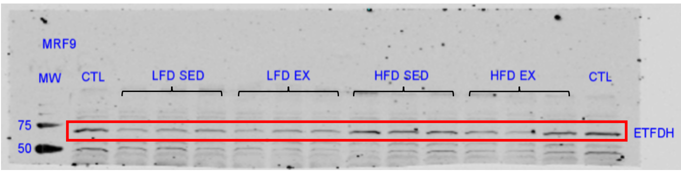

CPT – Blot MRF9 ~87 kDa 700 Channel

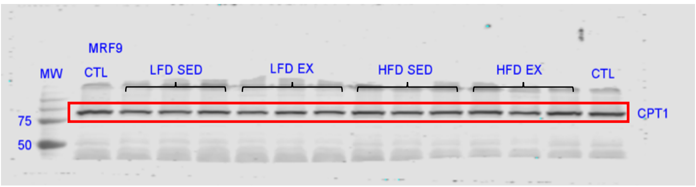

Figure 3B Mettl20 ~30kDa – Blot MRF19, 700 Channel

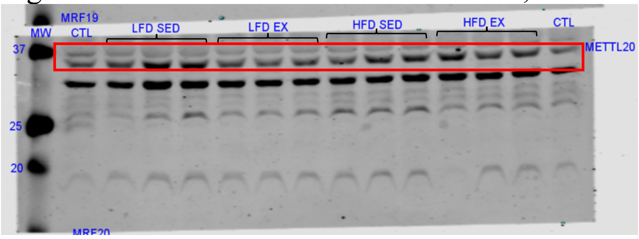

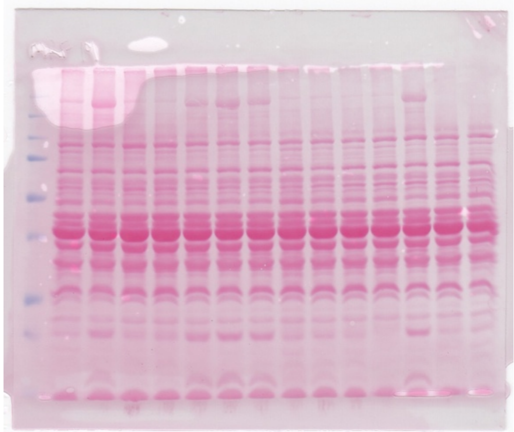

Figure 8A MFF ~26kDa – Blot MRF19, 700 Channel

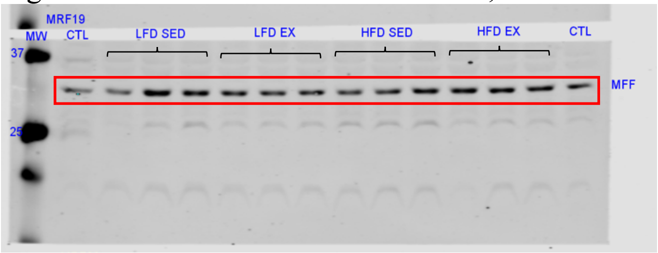

Figure 8A MFN2 ~78kDa – Blot MRF19, 800 Channel

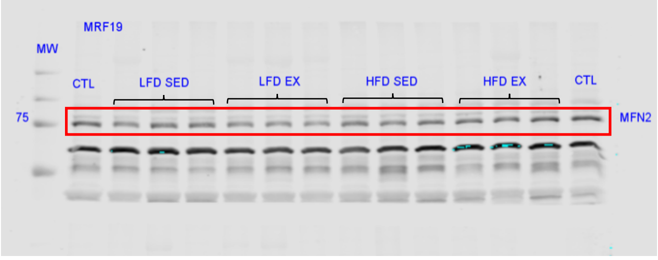

Figure 8A OPA1 ~80-100kDa Two Bands – Blot MRF19, 700 Channel

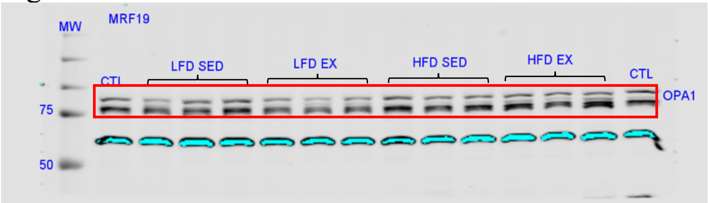

Figure 8C P62 ~62kDa – Blot MRF19, 700 Channel

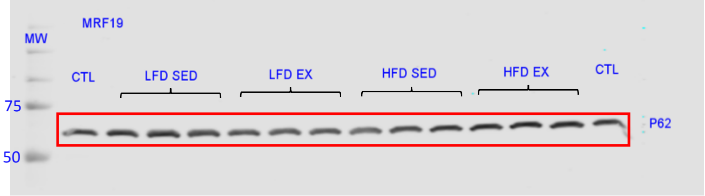

Figure 8A DRP1 ~78kDa – Blot MRF14, 800 Channel

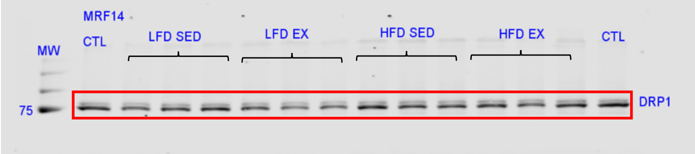

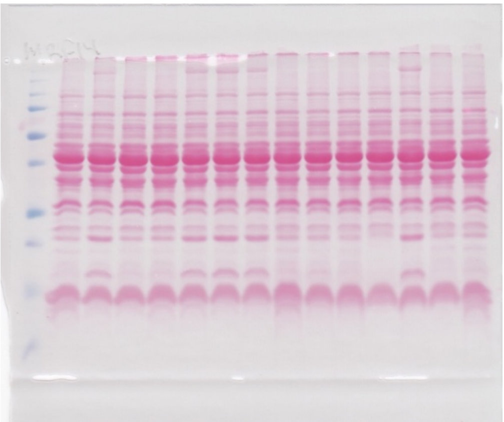

Figure 8C BCL2 ~25kDa – Blot MRF14, 800 Channel

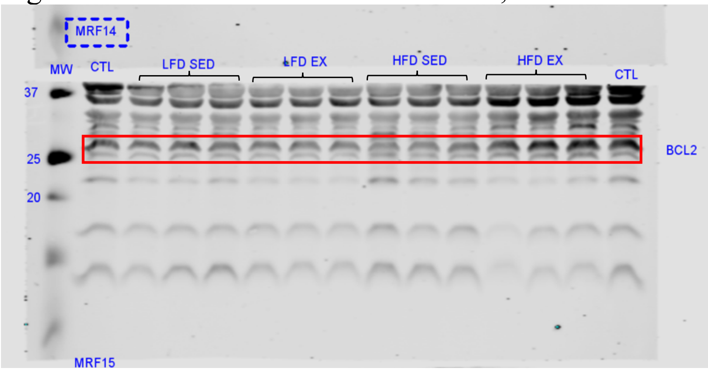

Figure 8C Parkin ~55kDa – Blot MRF14, 800 Channel

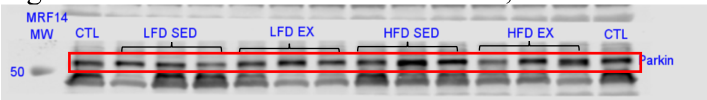

Figure 8C BNIP3 ~22-28kDa – Blot MRF14, 700 Channel

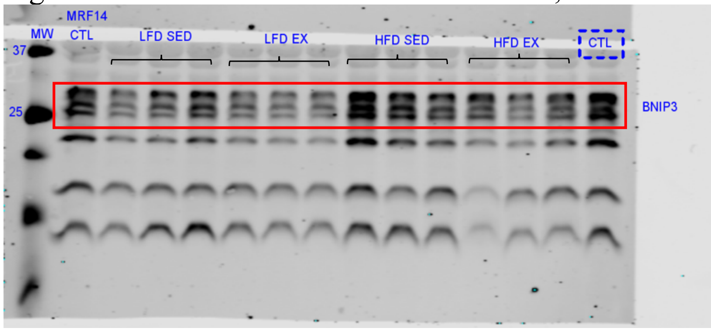

Figure 8C LC3II/I ~12/18kDa – Blot MRF14, 700 Channel

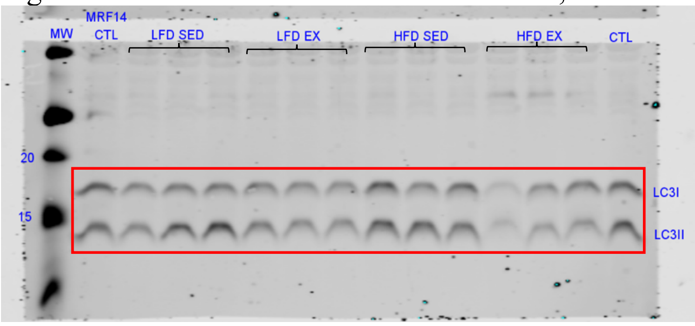

Figure 5A – OXPHOS; CI: 20kDa, CII: 30 kDa, CIII: 48 kDa, CIV: 40 kDa, CV: 55 kDa – Blot MRF4, 800 Channel

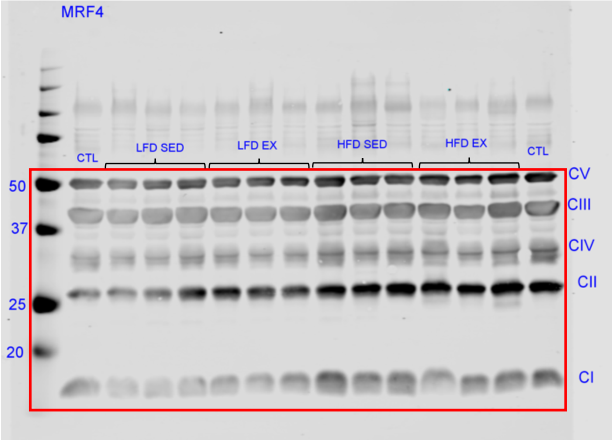

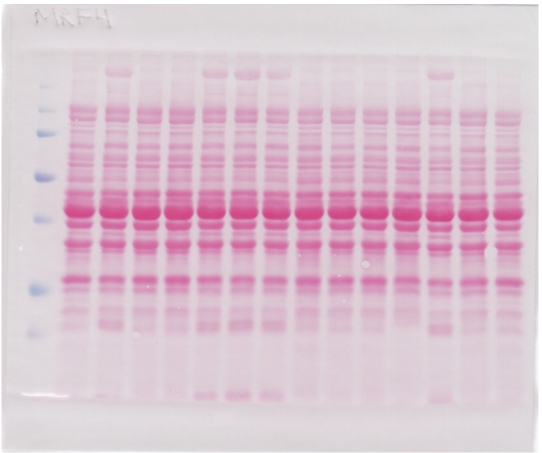

Figure 3C - HADH ~28 kDa – Blot MRF4, 700 Channel

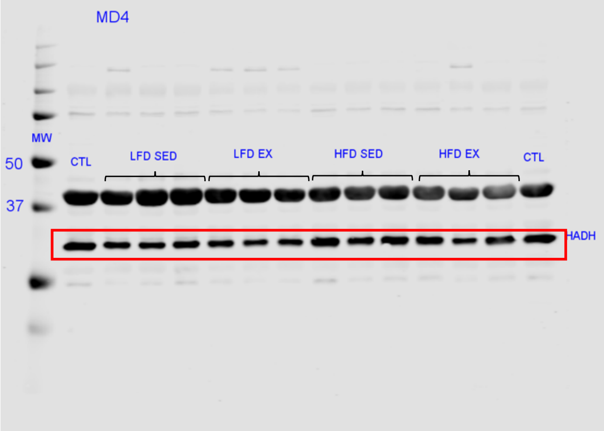

Figure 8C - Pink ~66 kDa – Blot MRF11, 700 Channel

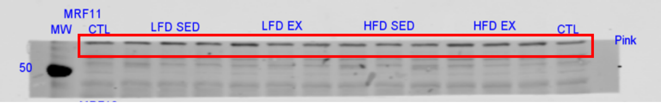

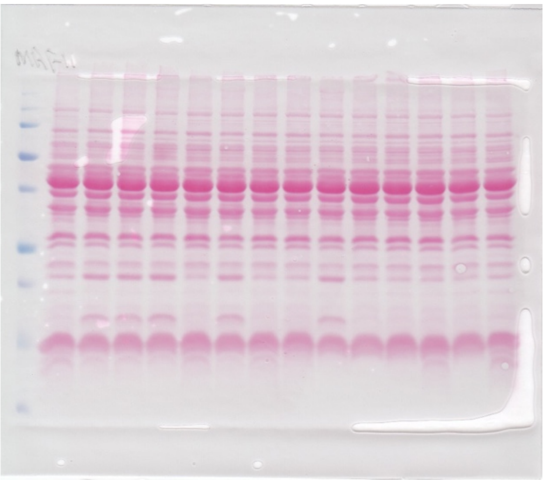

Figure 8E - Parkin ~66 kDa – Blot SSM4, 700 Channel

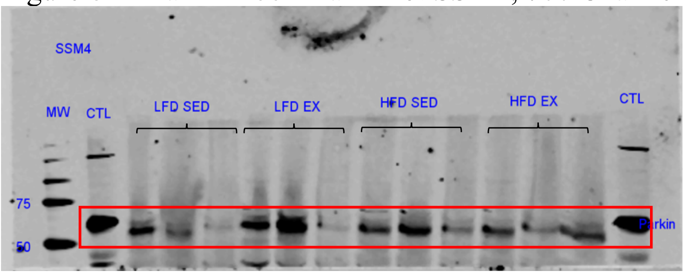

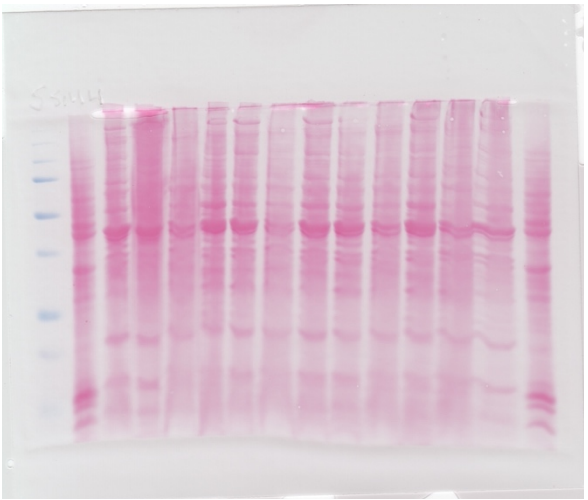

Figure 8E – BNIP3 ~22-28 kDa – Blot SSM4, 700 Channel

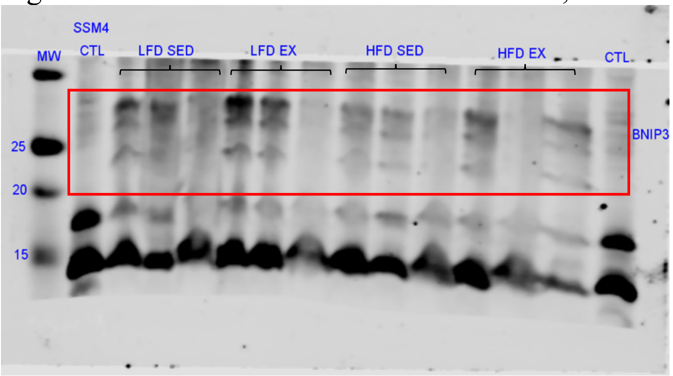

Figure 8E – P62 ~62 kDa – Blot SSM4, 700 Channel

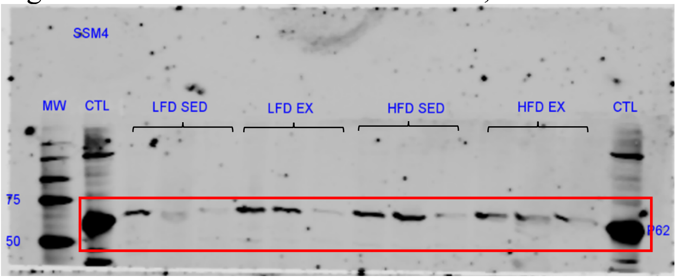

Figure 8E – LC3II ~12 kDa – Blot SSM4, 700 Channel

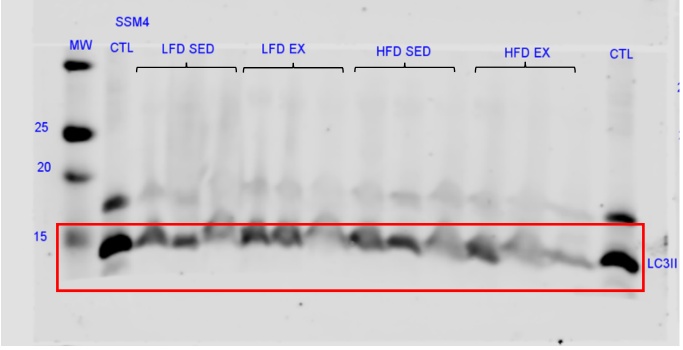

Figure 8E - Parkin ~66 kDa – Blot IMFM4, 700 Channel

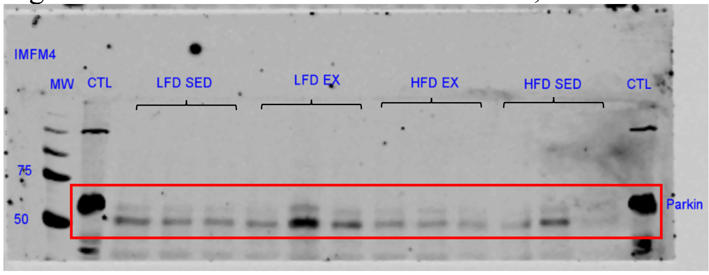

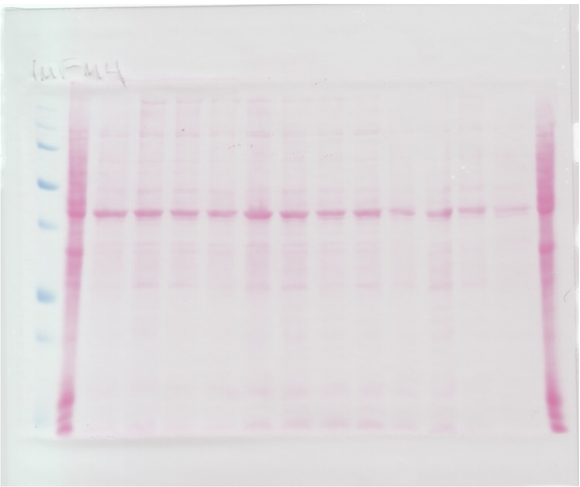

Figure 8E – BNIP3 ~22-28 kDa – Blot IMFM4, 700 Channel

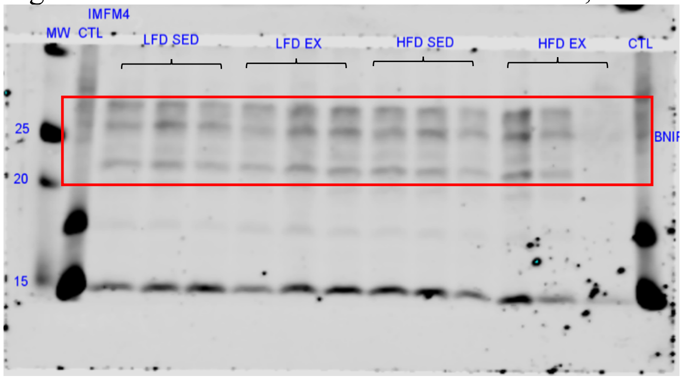

Figure 8E – P62 ~62kDa – Blot IMFM4, 700 Channel

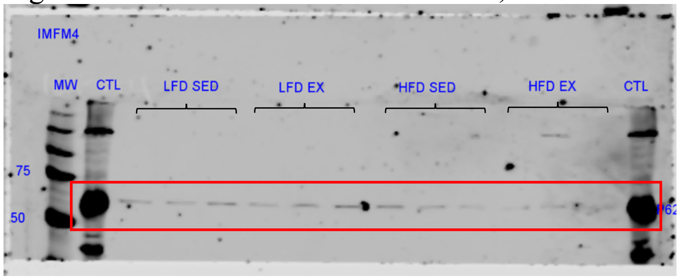

Figure 8E – LC3II ~12 kDa – Blot IMFM4, 700 Channel

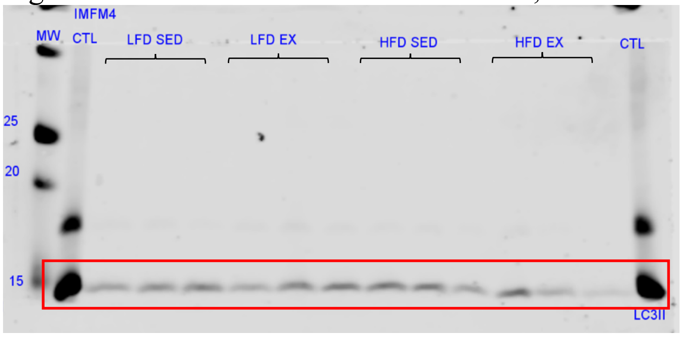

Supplement: Supplementary file 2 — Data S1. [file PHY2-11-e15840-s002.pdf]
